# Health determinants among participants in targeted health dialogues offered to all 40-year-old individuals in a metropolitan region of 1.4 million people

**DOI:** 10.1080/02813432.2024.2385547

**Published:** 2024-08-01

**Authors:** Emelie Stenman, Beata Borgström Bolmsjö, Anton Grundberg, Kristina Sundquist

**Affiliations:** a Center for Primary Health Care Research, Department of Clinical Sciences, Malmö, Lund University, Malmö, Sweden; b University Clinic Primary Care Skåne, Region Skåne, Sweden

**Keywords:** Cardiovascular risk factors, health behaviors, metabolic risk factors, primary health care, primary prevention

## Abstract

**Objective:**

To examine cardiovascular risk factors in 40-year-old participants in the health screening program targeted health dialogues (THDs).

**Design:**

Cross-sectional study.

**Setting:**

99 Swedish healthcare centers.

**Intervention:**

Metabolic risk factors and health behaviors were assessed. THDs were provided.

**Subjects:**

1831 (62.3%) THD participants that consented to take part in the research project.

**Main outcome measures:**

(1) Prevalence of metabolic risk factors (blood pressure, LDL cholesterol, fasting plasma glucose, BMI, waist-hip ratio) and unhealthy behaviors (tobacco, alcohol, diet, physical activity) by sex, education, and place of birth. (2) Associations between different health behaviors and between the number of unhealthy behaviors and prevalence of metabolic risk factors. (3) THD participation by sociodemographics compared to age-matched controls.

**Results:**

Men had a higher prevalence of all metabolic risk factors, excessive alcohol use and tobacco use than women. Lower educated individuals had a higher prevalence of metabolic risk factors (except for LDL cholesterol) and tobacco use than highly educated. Participants born outside Sweden had a higher prevalence of obesity, high waist-hip ratio, and tobacco use. Participants with 3–4 unhealthy behaviors had significantly higher prevalence of each of the metabolic risk factors except BMI. Women, highly educated and Swedish-born participants were slightly over-represented in the THDs.

**Conclusion:**

Considering the associations between unhealthy behaviors and metabolic risk factors, the THD method, covering lifestyle as well as objective health measures, may be an appropriate method for early identification of individuals at risk for future non-communicable diseases in the whole population with a specific focus on certain groups.

**ClinicalTrials.gov:**

NCT04912739

## KEY POINTS

The study presents health determinants in 40-year-old targeted health dialogue (THD) participants in a Swedish metropolitan, multi-ethnic region.

Only half of the men had normal blood pressure and less than 40% had a BMI < 25 kg/m^2^.

Having 3–4 unhealthy behaviors was associated with significantly higher prevalence of all metabolic risk factors (except BMI) compared to zero unhealthy behaviors.

The predominance of female, highly educated and Swedish-born participants in THDs calls for a targeted outreach to certain population groups.

## Introduction

Cardiovascular disease is one of the largest contributors to the global disease burden and the leading cause of death in many countries, including Sweden. Cancer is also a main cause of death worldwide and kills even more people than cardiovascular diseases in some high-income European countries [1,2]. However, both cardiovascular diseases and many types of cancer can, to a large extent, be prevented by improving modifiable risk factors, such as metabolic risk factors and poor health behaviors [3,4]. This provides an opportunity for prevention, both on an individual and societal level.

To encourage healthier lifestyles and prevent cardiovascular diseases, several regions in Sweden have implemented screening programs with targeted health dialogues (THDs) in primary care. These efforts commenced in the 1980s in the form of mixed individual and community intervention programs in two regions: the lifestyle health dialogues in Habo municipality [5] and the ‘Västerbotten Intervention Programme’, VIP, in the county of West Bothnia [6].

Previous evaluations of implemented THD programs have been reviewed by a Swedish expert group [7]. The results showed, with a moderate evidence level, that THDs have the potential to vastly reduce all-cause [5,8] and cardiovascular mortality [8]. There was also evidence that THDs can reduce cholesterol levels [9–11], fasting plasma glucose [12], systolic [9–12] and diastolic [9,10,12] blood pressure, BMI and waist circumference [9,13] as well as improve eating habits [9,13]. In addition, health economic evaluations have shown that the THD model is a cost-effective method in the health care sector [14].

Starting in 2020, THDs were implemented by the County Council in a pilot project among 40-year-olds in the southernmost region of Sweden, Scania, which includes the third largest metropolitan region in Sweden. In 2021, it was decided to invite all 40- and 50-year-olds in the region to a THD at their healthcare center. A research project was linked to the implementation, to create age-cohorts for long-term follow-up of cardiovascular risk factors and events.

**Figure 1.**
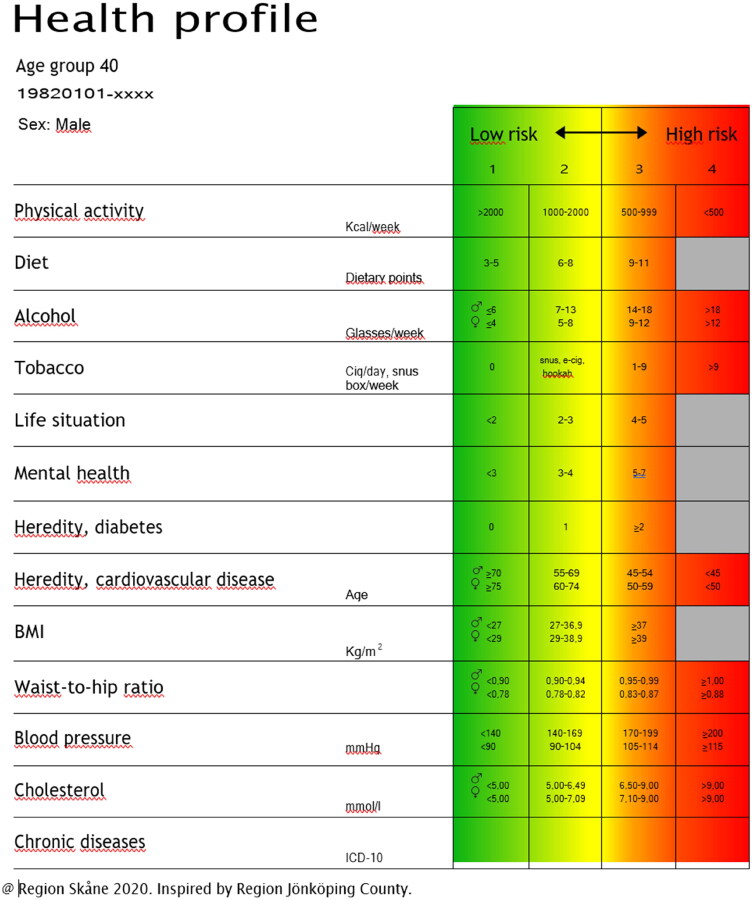
The health profile.

The measurements in the Scania THDs comprise a questionnaire about lifestyle and health, and a physical examination with blood sampling and anthropometric measures. The results are aggregated in an illustrative health profile with 13 categories (Figure 1). The health profile is used as a basis for counseling offered by specially trained healthcare personnel.

A novel contribution of the present THD study is that the model is applied in a metropolitan area. Another important contribution is that we took the place of birth into account, which has rarely been done in previous THD evaluations. Since Sweden has become a multiethnic country over time, especially in metropolitan areas, this increases the generalizability of our findings. In addition, we have used the comprehensive health profile to examine associations between different unhealthy behaviors, and between the number of unhealthy behaviors and metabolic risk factors.

The first aim was to examine metabolic risk factors and health behaviors in 40-year-old THD participants from a socioeconomically mixed population, originating from many different countries. Since most women undergo health examinations (including lifestyle) in connection with pregnancies, whereas men do not, it is possible that men in their 40s are unaware of their potential cardiovascular risk factors. Therefore, we included a focus on possible health differences between men and women. Since unhealthy behaviors tend to cluster [15] we hypothesized that this would be the case in our population as well. Thus, a second aim was to examine associations between different unhealthy behaviors, and also to test our hypothesis of associations between the number of unhealthy behaviors and prevalence of metabolic risk factors. A third aim was to examine whether participation in THDs differed by sociodemographic characteristics, i.e. do the THDs reach the most vulnerable population groups in a metropolitan region?

## Materials and methods

### Setting and study population

The Scania region is the southernmost county of Sweden with about 1.4 million inhabitants and a mix of rural and metropolitan areas. The region has approximately 170 primary healthcare centers, about half of which are public, with the other half being private. Both the public and the private centers are tax-financed and included in Sweden’s universal health care system where all inhabitants in the region are registered at a healthcare center.

The THD-model in Scania was designed and implemented by a THD method support team at the County Council. The team consists of general practitioners, nurses, dieticians, and physiotherapists that work in a central unit for disease prevention. They provide the healthcare center professionals with education and continuous method support regarding the THDs. The Center for Primary Health Care Research, a collaboration between Region Scania and Lund University, was invited to perform scientific studies and evaluations within the THD model. The present cross-sectional study was based on data derived from the measurements at the THDs. It included people who turned 40 years of age in 2021 or the first half of 2022 and took part in a THD at their healthcare center. The healthcare centers were instructed to invite all their registered 40-year-olds to a THD and all participants in the THDs were in turn invited to the research project. Thus, it was possible to participate in a THD without participating in the research project. Written informed consent was a prerequisite for taking part in the research project. Ethical approval was obtained from the Swedish Ethical Review Authority (registration number 2020-02689 with later amendments). Health parameters of the study population will be followed longitudinally and compared to the age-matched entire Swedish population as well as historical controls. Therefore, the study was registered at ClinicalTrials.gov, identifier: NCT04912739.

### The THD model

THD in Scania was adapted from the individual-based part of the lifestyle health dialogues in Habo municipality, which has been described in detail previously [16]. An invitation to a THD was sent out by letter to registered 40-year-olds from their healthcare center. This was followed up by a phone call to book the fasting blood test that preceded the health dialogue. Before the health dialogue, the participants also filled in an electronic questionnaire about family situation, place of birth, education, chronic diseases, family history of diseases, and health behaviors (tobacco, alcohol, diet, physical activity). On their first visit to the healthcare center, the participants underwent a fasting blood test to measure total cholesterol, low-density lipoprotein (LDL) cholesterol, high-density lipoprotein (HDL) cholesterol, and fasting plasma glucose. Body mass index (BMI), waist–hip ratio and blood pressure were also measured before the health dialogue. Blood pressure was measured in a sitting position after resting for five minutes. It was measured in both arms and at least two times with one minute in between. The mean value was registered. Around a week later, the participants went through a THD led by a specially trained health dialogue coach, i.e. a registered nurse, dietician, occupational- or physiotherapist, or physician with a 2-day education in the THD method and a 3-day education in motivational interviewing. At the THD, the health dialogue coach and participant together went through all the results from the questionnaire and measurements by using a visual tool—the health profile—which points out cardiovascular risk factors in 13 different categories that are grouped in (three or) four risk levels: green, yellow, orange, and red (Figure 1). Improvement opportunities regarding health behaviors were discussed. When necessary (e.g. suspicion of hypertension or blood test results outside the reference values), follow-up visits to the healthcare center were booked or referrals to other clinics were sent. Such clinical decisions were made based on a written, evidence-based method support provided to all health dialogue coaches.

### Data collection

All collected data in the THDs were stored in a quality register within the County Council of Scania. From there, pseudonymized data for those who had consented to take part in the research project were transferred to our research group. Data went through a monitoring process to ensure internal validity.

To examine if our study population was representative, aggregated data about sociodemographic characteristics (sex, educational level, and place of birth) for the entire population of 40-year-olds in Scania were collected from the Swedish government authority Statistics Sweden (2023)[17].

### Variables

Five metabolic risk factors were included in the analyses: blood pressure, LDL cholesterol, fasting plasma glucose, BMI, and waist-hip ratio. The health behaviors examined were physical activity, diet, alcohol, and tobacco, which are well-known to be involved in the etiology of non-communicable diseases [18]. Cut-offs for poor health behaviors were defined as not being ‘green’ in the health profile (Figure 1). The variables were categorized as described below.

Normal blood pressure was defined as <130/85 mmHg. High normal blood pressure was defined as a systolic blood pressure of 130–139 mmHg or a diastolic blood pressure of 85–89 mmHg. Suspicion of hypertension was defined as a systolic blood pressure ≥140 mmHg or a diastolic blood pressure ≥ 90 mmHg according to European guidelines [19]. Previously known hypertension was self-reported in the questionnaire and/or collected from the patient records, identified by ICD-codes I10-I13.

High cholesterol was defined as LDL cholesterol ≥ 5 mmol/L according to international guidelines [20].

High fasting plasma glucose level was defined as ≥ 6.1 mmol/L, which is the limit for impaired fasting glucose according to the WHO (2006). A suspicion of diabetes was defined as ≥7 mmol/L.

Overweight was defined as BMI ≥ 25 kg/m^2^ and obesity as BMI ≥ 30 kg/m^2^. In the dichotomized analyses, high BMI was defined as ≥ 25 kg/m^2^ according to the WHO (2021) since increased cardiovascular risk has been shown already at this level [21].

High waist–hip ratio was defined as ≥ 0.85 for women and ≥ 0.90 for men according to the WHO’s (2008) definition of abdominal obesity in the metabolic syndrome.

Insufficient physical activity was defined as burning less than 2000 kilocalories (kcal) per week in leisure time. The calculation was based on the question: *How physically active are you in your leisure time?* The question had four response alternatives: (1) sedentary leisure time, (2) moderate exercise, (3) strenuous exercise, (4) hard exercise. Participants who chose alternative 1 were directly categorized into the highest risk level in the health profile (<500 kcal/week), and participants who chose alternative 4 were directly categorized into the lowest risk level (>2000 kcal/week). Participants who chose alternative 2 or 3 were asked to respond to follow-up questions about the mode of transport taken to work and other leisure time physical activities. Physical activity was reported as minutes per week and season and multiplied with specific energy factors for different activities (range 0–10 points) [16].

The food questionnaire was a slightly adapted version of a previously validated questionnaire used in THDs in other Swedish regions [22]. Poor eating habits were calculated from a fat- and fiber index based on questions about the frequency of eating different kinds of food, and food quality. In addition, if the participants answered that they were having ‘sweets, chocolate, or sugar-sweetened drinks’ ≥ 2 times per day or ‘cakes or cookies’ ≥ 2 times per day, or both categories one time per day, they were moved one step to the right in the health profile, thus were considered as having increased risk.

Excessive alcohol intake was defined as drinking ≥ 5 (women) or ≥ 7 (men) standard glasses of alcohol (equivalent to 12–15 cl wine) per week. Drinking ≥ 4 (women) or ≥ 5 (men) standard glasses per occasion at least monthly was also categorized as excessive alcohol intake.

Tobacco use was defined as smoking cigarettes or using ‘snus’ (oral tobacco product), e-cigarettes or waterpipe regularly. In analyses of relationships, tobacco use was dichotomized into using/not using.

### Statistical analysis

Participant characteristics are presented as categories with numbers and percentages and for continuous variables, with means and standard deviations (SD). Differences in characteristics between sexes, level of education and place of birth were tested using Chi-squared tests for categorical variables and Welch’s *t*-tests for continuous variables. We also conducted sensitivity analyses using logistic and linear regression where sex, level of education and place of birth were all adjusted for each other.

To assess the associations between the four health behaviors, odds ratios (ORs) with 95% confidence interval (CI) were estimated using logistic regression, taking into account potential confounders including sex, education and place of birth. Differences in metabolic risk factors between those with at least three and no unhealthy behaviors were analyzed with Chi-squared tests or Fisher’s exact test. The chosen level of statistical significance was 0.05. All statistical analyses were done in R version 4.2.1 (R Core Team, 2022).

## Results

At the time of data collection, 99 healthcare centers of about 170 had started with THDs. At these healthcare centers, a total of 8479 40-year-olds were invited, 3985 accepted the invitation and 2937 completed their THD during the study period. Of these, 1831 gave consent to take part in the research project. Hence, the study participants constituted 21.6% of invited 40-year-olds, and 62.3% of those with completed health dialogues.

Data was handed over from a quality register collecting all data from the THDs. When the data was extracted, many invited participants had not yet had their THDs and therefore could not be included in the research project. Due to this, 1048 potential participants were excluded from the research project. A flowchart of the recruitment is shown in Supplementary Figure 1.

### Metabolic risk factors

Men had higher prevalences of all metabolic risk factors compared to women. 23.4% of men and 15.2% of women had high normal blood pressure, and 26.6% of men and 12.0% of women had a suspicion of hypertension. 8.0% of men and 2.4% of women had high LDL cholesterol. 10.0% of men and 5.0% of women had a fasting plasma glucose level that suggested impaired fasting glucose, and 3.3% of men and 0.8% of women had a fasting plasma glucose level that indicated suspected diabetes. These differences between men and women were all statistically significant (*p* < 0.001), also when using continuous variables (Table 1).

**Table 1.** Characteristics of THD participants.

Characteristic	Total	Women	Men	*p*-Value^a^
Sex, *n* (%)	1831 (100)	1010 (55.2)	821 (44.8)	<0.001
Level of education, *n* (%)				<0.001
≤9 years	107 (5.8)	48 (4.8)	59 (7.2)	
10–12 years	602 (32.9)	272 (26.9)	330 (40.2)	
>12 years	1120 (61.2)	689 (68.2)	431 (52.5)	
Missing	2 (0.1)	1 (0.1)	1 (0.1)	
Place of birth, *n* (%)				0.86
Sweden	1252 (68.4)	691 (68.4)	561 (68.3)	
Other European country	270 (14.7)	152 (15.0)	118 (14.4)	
Non-European country	309 (16.9)	167 (16.5)	142 (17.3)	
Blood pressure, *n* (%)				<0.001
Normal (<130/85)	1142 (62.4)	733 (72.6)	409 (49.8)	
High normal (130–139/85–89)	346 (18.9)	154 (15.2)	192 (23.4)	
Suspicion of hypertension (≥140/90)	339 (18.5)	121 (12.0)	218 (26.6)	
Missing	4 (0.2)	2 (0.2)	2 (0.2)	
Systolic blood pressure, mean (SD)	121.78 (13.38)	117.28 (11.79)	127.31 (13.14)	<0.001
Diastolic blood pressure, mean (SD)	78.61 (10.18)	76.82 (9.95)	80.81 (10.05)	<0.001
LDL cholesterol, *n* (%)				<0.001
Normal	1730 (94.5)	980 (97.0)	750 (91.4)	
High	90 (4.9)	24 (2.4)	66 (8.0)	
Missing	11 (0.6)	6 (0.6)	5 (0.6)	
LDL cholesterol, mean (SD)	3.29 (0.95)	3.06 (0.86)	3.57 (0.98)	<0.001
F-plasma glucose, *n* (%)				<0.001
≤6 mmol/l	1647 (90.0)	940 (93.1)	707 (86.1)	
6.1 − 6.9 mmol/l	133 (7.3)	51 (5.0)	82 (10.0)	
≥7 mmol/l	35 (1.9)	8 (0.8)	27 (3.3)	
Missing	16 (0.9)	11 (1.1)	5 (0.6)	
F-plasma glucose, mean (SD)	5.44 (0.79)	5.31 (0.51)	5.59 (1.01)	<0.001
BMI, *n* (%)				<0.001
<25	843 (46.0)	537 (53.2)	306 (37.3)	
25 − 29.9	642 (35.1)	282 (27.9)	360 (43.8)	
≥30	342 (18.7)	189 (18.7)	153 (18.6)	
Missing	4 (0.2)	2 (0.2)	2 (0.2)	
BMI, mean (SD)	26.28 (4.77)	25.89 (5.07)	26.75 (4.34)	<0.001
Waist-hip ratio, *n* (%)				<0.001
Normal	1092 (59.6)	739 (73.2)	353 (43.0)	
High	718 (39.2)	261 (25.8)	457 (55.7)	
Missing	21 (1.1)	10 (1.0)	11 (1.3)	
Physical activity, *n* (%)				0.59
Sufficient	579 (31.6)	314 (31.1)	265 (32.3)	
Insufficient	1252 (68.4)	696 (68.9)	556 (67.7)	
Eating habits, *n* (%)				0.66
Healthy	687 (37.5)	373 (36.9)	314 (38.2)	
Poor	1099 (60.0)	611 (60.5)	488 (59.4)	
Missing	45 (2.5)	26 (2.6)	19 (2.3)	
Alcohol consumption, *n* (%)				<0.001
Normal	1481 (80.9)	877 (86.8)	604 (73.6)	
Excessive	287 (15.7)	115 (11.4)	172 (21.0)	
Missing	63 (3.4)	18 (1.8)	45 (5.5)	
Tobacco use, *n* (%)				<0.001
None	1311 (71.6)	818 (81.0)	493 (60.0)	
Snus, e-cigarettes, hookah/waterpipe	286 (15.6)	70 (6.9)	216 (26.3)	
Cigarettes	233 (12.7)	121 (12.0)	112 (13.6)	
Missing	1 (0.1)	1 (0.1)	0 (0)	

^a^*p*-Value for comparison of sexes is determined using Chi-squared test for categorical variables and Welch’s *t*-test is used for continuous variables.

Regarding anthropometric measures, there were also sex differences. 43.8% of men and 27.9% of women were overweight (*p* < 0.001). 18.6% of men and 18.7% of women were obese. 55.7% of men and 25.8% of women had a high waist–hip ratio (*p* < 0.001) (Table 1).

Of participants with already known hypertension (*n* = 152), 67.1% of men and 43.2% of women still had an elevated blood pressure (≥140/90 mmHg) at the THD measurements (*p* = 0.002) (data not shown), thus did not have a well-controlled blood pressure, despite awareness of their condition.

Participants with ≤ 12 years education had a higher prevalence of elevated blood pressure (*p* = 0.001) and fasting plasma glucose (*p* = 0.002) compared to those with more than 12 years education. LDL cholesterol did not differ significantly between the educational groups when comparing dichotomized values, but when comparing continuous variables, participants with ≤ 12 years education had significantly higher LDL cholesterol. There were no significant differences between participants born in Sweden and outside Sweden regarding blood pressure, fasting plasma glucose or LDL cholesterol when comparing dichotomized values. However, when comparing continuous variables, participants born outside Sweden had lower systolic blood pressure, but higher LDL cholesterol compared to participants born in Sweden.

Participants with ≤ 12 years education had a higher prevalence of overweight and high waist–hip ratio compared to those with more than 12 years education (both *p* < 0.001). Participants born outside Sweden had a higher prevalence of overweight, obesity (*p* = 0.004), and high waist–hip ratio (*p* < 0.001), and higher BMI (*p* = 0.024) than their Swedish counterparts (Supplementary Table 1).

In a sensitivity analysis, we checked whether adjusting for sex, level of education and/or country of birth would affect the results regarding metabolic risk factors. All significant differences remained unchanged with two exceptions. Firstly, when adjusting for sex and level of education, the risk of elevated blood pressure became significantly lower among foreign-born compared to Swedish-born participants (*p* = 0.038). This is in line with the lower systolic blood pressure among foreign-born participants. Secondly, BMI was no longer significantly higher among foreign-born participants after adjusting for sex and level of education (*p* = 0.09) (data not shown).

### Health behaviors

Self-reported health behaviors are shown in Table 1. Barely a third (31.6%) of the participants were sufficiently physically active according to the health profile, i.e. expended >2000 kilocalories per week in their leisure time.

A total of 60% of the participants reported poor eating habits.

Regarding alcohol and tobacco consumption, there were sex differences. 21.0% of men and 11.4% of women had excessive alcohol consumption (*p* < 0.001). 39.9% of men were tobacco users; 26.3% used snus, e-cigarettes, or waterpipe, and 13.6% were cigarette smokers. The corresponding figures for women were 18.9, 6.9 and 12%, respectively (*p* < 0.001) (Table 1).

When comparing more than 12 years education with ≤12 years education, it turned out that participants with more than 12 years education had less tobacco use than those with lower education (*p* < 0.001) (Supplementary Table 1). When comparing participants born in Sweden versus foreign-born, those born in Sweden had less overall tobacco use (*p* < 0.001). Those born in Sweden more often used snus, e-cigarettes, or waterpipe (18.0 vs 10.5%), but foreign-born participants smoked cigarettes to a higher degree (21.2 vs 8.8%). Participants born outside Sweden had healthier eating habits and less alcohol consumption than those born in Sweden (both *p* < 0.001) (Supplementary Table 1). All significant differences in health behaviors remained unchanged after adjusting for sex, place of birth and/or level of education (data not shown).

### Associations between different health behaviors and between health behaviors and metabolic risk factors

A total of 1729/1831 participants had complete data about the four health behaviors. 182 (9.9%) of the participants had no unhealthy behaviors, 503 (27.5%) had one unhealthy behavior, 690 (37.7%) had two unhealthy behaviors, 293 (16.0%) had three unhealthy behaviors, and 61 (3.3%) had four unhealthy behaviors (data not shown). Thus, the majority had at least two unhealthy behaviors and almost 20% had at least three unhealthy behaviors.

Table 2 shows the associations between the four health behaviors. After adjusting for sex, level of education and place of birth, insufficient physical activity was significantly associated with poor eating habits and tobacco use. Tobacco use was, in addition to insufficient physical activity, also significantly associated with excessive alcohol consumption.

**Table 2.** Associations between health behaviors.

	Insufficient physical activity	Poor eating habits	Excessive alcohol use	Tobacco use
Insufficient physical activity				
Univariable	–	1.78 (1.45–2.18)	0.86 (0.66–1.13)	1.31 (1.05–1.64)
Multivariable	–	**1.83 (1.49–2.25)**	0.90 (0.69–1.19)	**1.31 (1.04–1.65)**
Poor eating habits				
Univariable	1.78 (1.45–2.18)	–	1.14 (0.88–1.49)	1.09 (0.88–1.35)
Multivariable	**1.83 (1.49–2.25)**	–	1.06 (0.81–1.39)	1.10 (0.88–1.38)
Excessive alcohol use				
Univariable	0.86 (0.66–1.13)	1.14 (0.88–1.49)	–	3.06 (2.36–3.98)
Multivariable	0.90 (0.69–1.19)	1.06 (0.81–1.40)	–	**2.98 (2.25–3.93)**
Tobacco use				
Univariable	1.31 (1.05–1.64)	1.09 (0.88–1.35)	3.06 (2.36–3.98)	–
Multivariable	**1.30 (1.03–1.65)**	1.10 (0.88–1.38)	**3.01 (2.28–3.99)**	–

Data are presented as odds ratios (95% CI).

Multivariable models are adjusted for sex, level of education and place of birth.

Bold values denote significant associations in the multivariable analysis.

**Figure 2.**
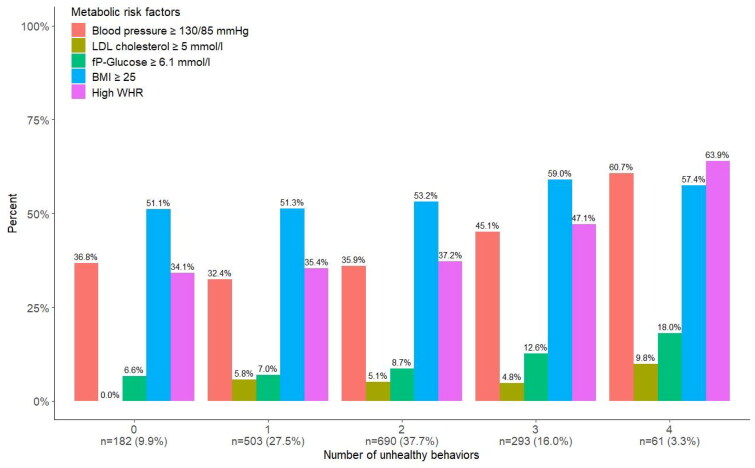
Proportions of metabolic risk factors by number of unhealthy behaviors.

When comparing those who had three or more unhealthy behaviors with those who had no unhealthy behaviors, we found that they had a higher prevalence of all metabolic risk factors (though non-significant for BMI): blood pressure ≥130/85 mmHg (48.2 vs 36.8%; *p* < 0.013), high LDL cholesterol (6.0 vs 0%; *p* < 0.001) high fasting plasma glucose (14.1 vs 6.6%; *p* < 0.001), high waist–hip ratio (51.2 vs 34.1%; *p* < 0.001), and BMI ≥ 25 (59.6 vs 51.1%; *p* = 0.053). Figure 2 shows the proportions of metabolic risk factors by number of unhealthy behaviors.

### Participation in THDs by sociodemographic characteristics

Among the research participants, 55.2% were women, 61.2% had more than 12 years education, and 68.4% were born in Sweden (Table 1). The proportion who had more than 12 years of education differed significantly between participants born in Sweden and participants born outside Sweden (64.0 and 55.1%, respectively) (Supplementary Table 1).

**Figure 3.**
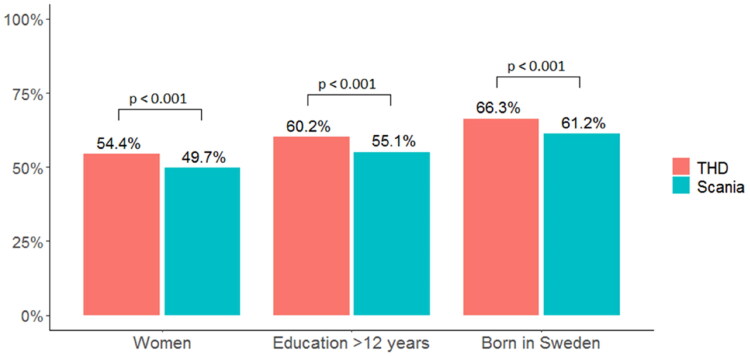
Percentage of women, participants with >12 years education and Sweden-born participants in the THDs compared with the aged-matched population in Scania.

Among all THD participants (regardless of research participation; aggregated data), 54.4% were women, which can be compared to 49.7% women among all Scania residents born in 1981 and 1982. Of the THD participants, 60.2% had more than 12 years of education. The corresponding proportion among Scania residents in the same age group were 55.1%. 66.3% of the THD-participants were born in Sweden, which can be compared to 61.2% of Scania residents in the same age group. The differences in sociodemographic characteristics were small but significant (Figure 3; [17]). Thus, THDs seem to attract slightly more women, people with high education and people born in Sweden compared with the age-matched population in Scania.

## Discussion

Targeted health dialogues (THDs) are currently being introduced at around 170 healthcare centers in Sweden’s southernmost region, Scania. The goal is to invite all 40- and 50-year-old residents to a THD in order to prevent future cardiovascular diseases, diabetes and other non-communicable diseases. This is becoming the most extensive implementation of THDs in Sweden, and to continuously evaluate the model’s expediency and effect, a research project was connected from start in the form of a prospective cohort study with a unique biobank. As THDs have been evaluated before, the novelty of the present study lies above all in the setting and timing—a multi-ethnic cohort of 40-year-olds in a large, metropolitan region, in the 2020s. It also provides a study protocol and reference values for future research. So, how healthy were the 40-year-olds in Scania? It turned out that there may be improvement opportunities, especially among men. In addition, the predominance of female, highly educated and Swedish-born participants in the THDs calls for a targeted outreach to other population groups.

In general, men had more metabolic risk factors than women—seven out of the nine examined risk factors were significantly more frequent in men. Some of these had strikingly high prevalence. For example, more than 60% were overweight or obese and more than 50% had a high waist–hip ratio. The high levels of overweight/obesity and high waist–hip ratio are worrying. Overweight is a risk factor for cardiovascular disease [23], and visceral obesity is the central component of the metabolic syndrome [24]. The other criteria of the metabolic syndrome (dyslipidemia, high blood glucose and high blood pressure), of which at least two should be present [24]) were also common, especially in men. Half of the men had a blood pressure ≥130/85 mmHg, one fourth had suspicion of hypertension (≥140/90 mmHg), and 13% had a fasting plasma glucose ≥6.1 mmol/l. The high share of men having signs of prehypertension or prediabetes points towards the importance of primary prevention, where participants can be informed of their possibility of reducing the cardiovascular risk through lifestyle modification. This could improve public health via informed decisions about lifestyle.

Biological mechanisms may explain part of the sex differences. For example, men and women store body fat in diverse ways, with different cardiometabolic characteristics. There are hormonal mechanisms involved in glucose and lipid metabolism and it has been shown that the metabolic syndrome is less common in premenopausal women but more common in postmenopausal women compared to men [25]. Nevertheless, our results stress the importance of reaching out to men when inviting individuals to participate in THDs.

The results regarding health behaviors can be put into perspective by comparisons with the Scania public health survey [26] and the Swedish national health survey [27]. Among THD participants, just over 30% reported sufficient physical activity (i.e. expended > 2000 kcal per week in leisure time), whereas in the Scania public health survey, over 60% among 30–44-year-olds were sufficiently active, and in the national public health survey, over 70% among 30–44-year-olds were sufficiently active (≥150 activity minutes per week). Sufficient physical activity in our study corresponded to a relatively rigorous threshold. However, considering the high levels of metabolic risk factors, e.g. overweight and obesity, it may be better to keep the high threshold in the health profile to ensure that physical activity gets enough attention in the health dialogue.

37.5% Of the THD participants reported healthy eating habits. This is in the similar range, but slightly higher, compared to the National public health survey, in which 34% reported at least two daily servings of vegetables/root vegetables (healthy food indicator) [27]. These results indicate that a large percentage need to improve their eating habits to reduce their cardiovascular risk.

A total of 15.7% of the THD participants reported excessive alcohol consumption, which can be compared to 11% among 30–44-year-olds in the Scania public health survey and 13.4% among 30–44-year-olds in the national public health survey. The threshold for excessive alcohol consumption was slightly lower in the THDs than in the public health surveys that use the Alcohol Use Disorders Identification Test (AUDIT-C) as a template [28]. A recent systematic review by the Global Burden of Disease (GBD), suggests, though, that the guidelines and recommendations need to be revised and that the current thresholds are too high for younger populations in all regions [29]. Thus, it may be appropriate to keep the threshold in the health profile, again to ensure that the question on alcohol gets enough attention in the health dialogue.

12.7% of the THD participants were cigarette smokers (daily or sometimes), which can be compared to 8.9% among 30–44-year-olds in the national public health survey [27]. In the THDs, participants born outside Sweden had a much higher prevalence of smoking than participants born in Sweden. This highlights the importance of reaching out with THDs to foreign-born residents, considering the highly detrimental effects of tobacco smoking being one of the major risk factors for cardiovascular and all-cause mortality [30].

All four health behaviors were partly related to each other. As has been shown previously [18], excessive alcohol consumption and tobacco use seem to cluster with almost threefold odds of having one if the other is present. Two different types of clustering have been suggested, addictive (e.g. smoking and alcohol) and health promoting (e.g. physical activity and healthy eating habits) [31]. A similar pattern was observed in our study, with the exception that low physical activity, in addition to poor eating habits, was associated with increased odds of tobacco use.

Almost 20% of the THD participants had at least three unhealthy behaviors and they had higher prevalences of all metabolic risk factors (non-significant for BMI) compared to those with no unhealthy behaviors. It is also noteworthy that less than 50% had normal BMI, irrespective of number of unhealthy behaviors, and among those with no or only one unhealthy behavior one third still had a blood pressure above normal. A Spanish study of the same four health behaviors showed a similar distribution regarding the number of health behaviors and found that those with three to four unhealthy behaviors also had higher odds of poor self-rated health [15]. In a following study by the same authors, they also found higher odds of non-compliance to different kinds of health screening for those with a high number of unhealthy behaviors (although causality could not be assessed) [32]. This brings us to the importance of reaching the most vulnerable population groups.

The THD participants were not entirely representative of the total age-matched population in terms of sex, educational level, and place of birth; it turned out that the groups with more risk factors were slightly under-represented. As discussed, men had more metabolic risk factors than women, and men with already-known hypertension were less well-regulated. Participants with ≤12 years education had more metabolic risk factors than those with longer education, and although participants born outside Sweden had healthier eating habits and less alcohol use, they had much higher prevalence of cigarette smoking, obesity and high waist–hip ratio (the significance regarding higher BMI in foreign-born participants disappeared when adjusting for sex and level of education, suggesting that other factors than place of birth may explain parts of the differences). Non-participants in THDs have been described before. In the ‘Västerbotten Intervention Programme’, individuals who were single, had low income or were born outside Sweden tended to participate to a lower extent compared to those who were married/cohabitating, had medium to high income or were born in Sweden [33]. Non-participants in the lifestyle health dialogues in Habo municipality had higher healthcare consumption, were more often living alone, smoked, were unemployed, and registered at the social welfare office or temperance board. Common reasons for not attending THDs in Habo included having recently attended another health examination or not being interested [34]. Participation in a THD is voluntary, and the interest may differ between groups. But since health equity is a pillar of the Swedish healthcare system, and there is evidence that THDs can improve health and prevent both all-cause and cardiovascular mortality, efforts are needed to optimize the outreach. Therefore, Scania County Council is planning an information campaign that targets underrepresented groups.

### Strengths and limitations

A strength of the study is its mixed population in a metropolitan area with THD participants from different socioeconomic conditions and countries of origin. Another strength is the comprehensive questionnaire and physical examination that provides a vast amount of new data on cardiovascular risk factors among relatively young participants. One limitation of the study is that less than half of the 40-year-olds that were invited to a THD accepted the invitation. This may affect the external validity. Other limitations of the study include the predetermined design of the questionnaire and examinations. Some questions and measures are not entirely comparable with national or international standards, e.g. triglycerides were not measured, which complicates the estimates of the metabolic syndrome. The reason for not measuring triglycerides is that the THDs constitute a first, relatively broad screening. When dyslipidemia is detected, the participant is referred for further examinations, including triglyceride measurement (ref. personal communication with the THD method support team in Region Scania).

Furthermore, BMI was included among the risk variables, which may seem redundant since it is interrelated with waist–hip ratio. However, the study population was relatively young, and BMI was considered an important marker for the risk of developing metabolic syndrome in the future. BMI is also a risk factor for cardiovascular disease in itself [23].

The health behaviors were self-reported, which implies a risk of bias. In addition, some definitions can be questioned, e.g. in the health profile categories physical activity and eating habits, which both rely on questionnaires that may need to be adapted to a more modern and multiethnic lifestyle [22]. An additional limitation is the cross-sectional study design that prevents analyses of causality.

## Conclusion

Men had more metabolic risk factors than women, and participants with ≤12 years of education had more metabolic risk factors compared to those with longer education. Participants born outside Sweden were cigarette smokers and obese to a higher degree than participants born in Sweden.

Considering that only half of the men had normal blood pressure, less than 40% had normal BMI, and the prevalence of unhealthy behaviors was high, a large share of the male and relatively young population may be on a slippery slope towards developing cardiovascular complications. The women were not far behind. The current situation suggests an urgent public health problem that warrants action, e.g. in the form of preventive interventions such as the THDs, targeting the whole population with individualized health advice and timely medical treatment as needed. The present study constitutes the first part of a longitudinal research project collecting a comprehensive amount of health data including body composition measures and biobank samples. The research project has the potential to add new important insights into risk factors and mechanisms behind cardiovascular disease, other diseases, and premature death in a relatively young, multi-ethnic population.

## Supplementary Material

Supplementary Figure 1 and Table 1 240702.docx
