# Supplementary material for: Health determinants among participants in targeted health dialogues offered to all 40-year-old individuals in a metropolitan region of 1.4 million people
Source: Scand J Prim Health Care. 2024 Aug 1;43(1):24–35. doi: 10.1080/02813432.2024.2385547 (PMC11834821; doi:10.1080/02813432.2024.2385547)
Supplement: Supplementary Figure 1 and Table 1 240702.docx [file IPRI_A_2385547_SM8331.docx]

Supplementary Figure 1. Recruitment of research participants.


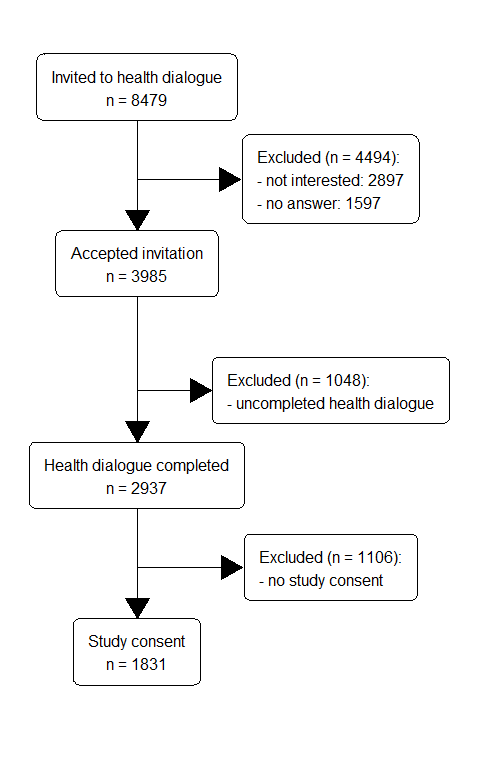


Supplementary Table 1. Baseline characteristics broken down by educational level and place of birth.

|  | **Level of education** | | | **Place of birth** | | |
| --- | --- | --- | --- | --- | --- | --- |
| **Characteristic** | **≤ 12 years** | **> 12 years** | **p-value^a^** | **Sweden** | **Outside Sweden** | **p-value^b^** |
| Level of education, n (%) |  |  | - |  |  | < 0.001 |
| ≤ 9 years | - | - |  | 36 (2.9) | 71 (12.3) |  |
| 10 – 12 years | - | - |  | 415 (33.1) | 187 (32.3) |  |
| > 12 years | - | - |  | 801 (64.0) | 319 (55.1) |  |
| Missing | - | - |  | 0 (0) | 2 (0.3) |  |
| Place of birth, n (%) |  |  | 0.001 |  |  | - |
| Sweden | 451 (63.6) | 801 (71.5) |  | - | - |  |
| Other European country | 116 (16.4) | 154 (13.8) |  | - | - |  |
| Non-European country | 142 (20.0) | 165 (14.7) |  | - | - |  |
| Blood pressure, n (%) |  |  | < 0.001 |  |  | 0.16 |
| Normal (<130/85) | 407 (57.4) | 734 (65.5) |  | 764 (61.0) | 378 (65.3) |  |
| High normal (130-139/85-89) | 143 (20.2) | 203 (18.1) |  | 240 (19.2) | 106 (18.3) |  |
| Suspicion of hypertension (≥140/90) | 159 (22.4) | 179 (16.0) |  | 245 (19.6) | 94 (16.2) |  |
| Missing | 0 (0) | 4 (0.4) |  | 3 (0.2) | 1 (0.2) |  |
| Systolic blood pressure, mean (SD) | 123.60 (13.61) | 120.64 (13.09) | < 0.001 | 122.69 (13.02) | 119.80 (13.92) | < 0.001 |
| Diastolic blood pressure, mean (SD) | 79.66 (10.48) | 77.94 (9.94) | < 0.001 | 78.63 (9.82) | 78.57 (10.93) | 0.92 |
| LDL cholesterol, n (%) |  |  | 0.38 |  |  | 0.20 |
| Normal | 668 (94.2) | 1060 (94.6) |  | 1189 (95.0) | 541 (93.4) |  |
| High | 39 (5.5) | 51 (4.6) |  | 56 (4.5) | 34 (5.9) |  |
| Missing | 2 (0.3) | 9 (0.8) |  | 7 (0.6) | 4 (0.7) |  |
| LDL cholesterol, mean (SD) | 3.40 (0.97) | 3.22 (0.93) | < 0.001 | 3.25 (0.94) | 3.38 (0.96) | 0.008 |
| F-plasma glucose, n (%) |  |  | 0.002 |  |  | 0.11 |
| ≤ 6 mmol/l | 618 (87.2) | 1028 (91.8) |  | 1139 (91.0) | 508 (87.7) |  |
| 6.1 – 6.9 mmol/l | 69 (9.7) | 63 (5.6) |  | 81 (6.5) | 52 (9.0) |  |
| ≥ 7 mmol/l | 17 (2.4) | 18 (1.6) |  | 22 (1.8) | 13 (2.2) |  |
| Missing | 5 (0.7) | 11 (1.0) |  | 10 (0.8) | 6 (1.0) |  |
| F-plasma glucose, mean (SD) | 5.54 (1.04) | 5.37 (0.57) | < 0.001 | 5.42 (0.84) | 5.48 (0.65) | 0.08 |
| BMI, n (%) |  |  | < 0.001 |  |  | 0.004 |
| < 25 | 258 (36.4) | 585 (52.2) |  | 606 (48.4) | 237 (40.9) |  |
| 25 – 29.9 | 274 (38.6) | 366 (32.7) |  | 430 (34.3) | 212 (36.6) |  |
| ≥ 30 | 177 (25.0) | 165 (14.7) |  | 213 (17.0) | 129 (22.3) |  |
| Missing | 0 (0) | 4 (0.4) |  | 3 (0.2) | 1 (0.2) |  |
| BMI, mean (SD) | 27.31 (4.92) | 25.62 (4.56) | < 0.001 | 26.11 (4.82) | 26.64 (4.65) | 0.024 |
| Waist-hip ratio, n (%) |  |  | < 0.001 |  |  | < 0.001 |
| Normal | 346 (48.8) | 744 (66.4) |  | 785 (62.7) | 307 (53.0) |  |
| High | 351 (49.5) | 367 (32.8) |  | 460 (36.7) | 258 (44.6) |  |
| Missing | 12 (1.7) | 9 (0.8) |  | 7 (0.6) | 14 (2.4) |  |
| Physical activity, n (%) |  |  | 0.15 |  |  | 0.08 |
| Sufficient | 210 (29.6) | 368 (32.9) |  | 412 (32.9) | 167 (28.8) |  |
| Insufficient | 499 (70.4) | 752 (67.1) |  | 840 (67.1) | 412 (71.2) |  |
| Eating habits, n (%) |  |  | 0.08 |  |  | < 0.001 |
| Healthy | 249 (35.1) | 436 (38.9) |  | 438 (35.0) | 249 (43.0) |  |
| Poor | 445 (62.8) | 654 (58.4) |  | 790 (63.1) | 309 (53.4) |  |
| Missing | 15 (2.1) | 30 (2.7) |  | 24 (1.9) | 21 (3.6) |  |
| Alcohol consumption, n (%) |  |  | 0.18 |  |  | < 0.001 |
| Normal | 556 (78.4) | 923 (82.4) |  | 965 (77.1) | 516 (89.1) |  |
| Excessive | 120 (16.9) | 167 (14.9) |  | 237 (18.9) | 50 (8.6) |  |
| Missing | 33 (4.7) | 30 (2.7) |  | 50 (4.0) | 13 (2.4) |  |
| Tobacco use, n (%) |  |  | < 0.001 |  |  | < 0.001 |
| None | 432 (60.9) | 877 (78.3) |  | 917 (73.2) | 394 (68.0) |  |
| Snus, e-cigarettes, waterpipe | 144 (20.3) | 142 (12.7) |  | 225 (18.0) | 61 (10.5) |  |
| Cigarettes | 133 (18.8) | 100 (8.9) |  | 110 (8.8) | 123 (21.2) |  |
| Missing | 0 (0) | 1 (0.1) |  | 0 (0) | 1 (0.2) |  |

^a^ P-value for comparison of level of education is determined using Chi-squared test for categorical variables and Welch’s t-test for continuous variables.
^b^ P-value for comparison of place of birth is determined same as ^a^.
